# Selective decontamination using antibiotics in ICU patients: counterfactual protection versus contextual hazard toward bacteremia incidences

**DOI:** 10.1186/cc14007

**Published:** 2014-12-03

**Authors:** JC Hurley

**Affiliations:** 1 Melbourne Medical School, University of Melbourne and Ballarat Health Services, Ballarat, Australia

## Introduction

Among methods for preventing pneumonia and possibly also bacteremia in ICU patients, selective digestive decontamination (SDD; topical with or without protocolized parenteral antibiotic) appears most effective within randomized concurrent controlled trials (RCCTs) [1]. However, whether parenteral antibiotic is required, and whether SDD actually increases pneumonia incidences in SDD RCCTs versus the broader ICU pneumonia evidence base, remain unresolved [2,3]. The purpose of this analysis is to test for counterfactual and contextual effects of the topical and parenteral SDD components on the bacteremia incidence versus the broader evidence base related to the patient group at risk of VAP.

## Methods

Bacteremia incidence proportion data were extracted from component (control and intervention) groups from studies investigating antibiotic (SDD) or nonantibiotic methods of VAP prevention. Both the counterfactual and the contextual effects of SDD factorized as topical or protocolized parenteral exposures were estimated using random-effects meta-analysis of study and group level data. Studies without any prevention methods under study constituted the reference category (benchmark groups).

## Results

As a counterfactual within RCCTs, SDD when given as combined topical and parenteral antibiotic appears to halve the bacteremia incidence (odds ratio (OR) 0.59; 0.48 to 0.73; *n *= 18 studies). As a contextual however, the mean bacteremia incidence among 27 control groups (17.1%; 13.1 to 22.1%) and 12 intervention groups receiving topical antibiotic alone (16.2%; 9.1 to 27.3%) from SDD RCCTs is double that of 36 benchmark groups (8.3; 7.0 to 10.8%) and 19 control groups from studies of nonantibiotic methods (7.7%; 5.2 to 11.1). The upward dispersion in bacteremia incidence among component groups from SDD RCCTs away from this benchmark is striking with all but two of the 27 control groups and all but two of 12 SDD intervention groups that did not receive PPAP being above this benchmark.

## Conclusion

The major contextual hazard of SDD toward bacteremia among ICU patients is inapparent within individual studies. The apparent protection in SDD RCCTs is spurious as the SDD counterfactual is conflated by the strong contextual effect with partial mitigation by SDD protocolized parenteral antibiotic. Not only is the safety of SDD within the ICU environment unclear, but this SDD contextual effect may conflate the apparent SDD counterfactual effect on the incidence of bacteremia, as with VAP.
